# Development and Initial Psychometric Validation of a Nurse‐Assessed Discharge Readiness Scale for Patients With Coronary Heart Disease Following Percutaneous Coronary Intervention

**DOI:** 10.1155/jonm/5080347

**Published:** 2026-10-19

**Authors:** Xingxing Mo, Ting Liang, Juejin Li, Xuan Wang

**Affiliations:** ^1^ Department of Cardiology Sichuan Provincial People’s Hospital School of Medicine University of Electronic Science and Technology of China Chengdu China uestc.edu.cn; ^2^ Department of Nursing Sichuan Provincial People’s Hospital School of Medicine University of Electronic Science and Technology of China Chengdu China uestc.edu.cn

**Keywords:** coronary heart disease, discharge readiness, instrument development, percutaneous coronary intervention, psychometric validation, transitional care

## Abstract

**Background:**

Following percutaneous coronary intervention (PCI), patients with coronary heart disease (CHD) face multiple disease‐ and treatment‐related self‐management demands after discharge. Accurate assessment of discharge readiness is essential for identifying areas in which patients may require additional discharge education and transitional care support. The widely used Readiness for Hospital Discharge Scale (RHDS) assesses overall discharge readiness across four domains: personal status, knowledge, coping ability and expected support. As a generic instrument, however, its broadly framed items may not adequately capture PCI‐specific aspects of readiness, including treatment adherence, puncture‐site healing, recognition of warning symptoms and preparedness for cardiac rehabilitation.

**Objective:**

To develop and initially validate a nurse‐assessed Discharge Readiness Scale for Patients with CHD Following PCI (DRS‐CHD‐PCI).

**Methods:**

A multistage instrument development and validation study was conducted. Candidate items were generated using transition theory, a targeted review of relevant literature, clinical guidelines and existing discharge‐readiness instruments, and semistructured interviews with patients with CHD following PCI and with healthcare professionals. Items were refined through two rounds of Delphi expert consultations and a pilot test with 20 patients. During psychometric validation, nurses assessed 444 patients on the planned discharge day; assessments took approximately 10–15 min. The sample was randomly split into two subsamples for exploratory and confirmatory factor analyses. Psychometric evaluation included item analysis, content validity, structural validity, convergent and discriminant validity, internal consistency and split‐half reliability.

**Results:**

The final scale comprised 17 items across five dimensions. Exploratory factor analysis supported a five‐factor structure that explained 75.84% of the total variance. Both the first‐ and second‐order confirmatory factor analysis models showed acceptable fit. Cronbach’s *α* was 0.945 for the total scale and ranged from 0.813 to 0.894 across the five dimensions.

**Conclusions:**

The DRS‐CHD‐PCI is a 17‐item, five‐dimensional scale with initial evidence supporting its content validity, structural validity and internal consistency. Further multicentre longitudinal studies are needed to evaluate its predictive validity and clinical utility and to determine how its scores can be meaningfully interpreted in clinical practice.

**Implications for Nursing Management:**

The DRS‐CHD‐PCI may assist nursing managers and cardiovascular nursing teams by providing a structured, assessment of discharge readiness before discharge and informing nurse‐led transitional care planning following PCI. Lower total or dimension scores may highlight areas in which patients require further clinical assessment, additional education, or follow‐up.

## 1. Introduction

Coronary heart disease (CHD) is a major contributor to the global burden of cardiovascular disease. According to the World Health Organization, cardiovascular diseases remain the leading cause of death worldwide, accounting for an estimated 19.8 million deaths in 2022, or approximately 32% of all global deaths, with around 85% of cardiovascular deaths attributable to heart attacks and strokes [1]. In China, the burden of CHD remains substantial. According to the Report on Cardiovascular Health and Diseases in China 2024, the prevalence of CHD among adults aged 18 years and above was 758 per 100,000 [2]. Percutaneous coronary intervention (PCI) is an important revascularisation strategy for CHD, relieving symptoms of myocardial ischaemia caused by coronary artery stenosis and improving clinical outcomes in patients with appropriate indications [3, 4]. However, PCI does not eliminate the underlying pathological processes or risk factors of CHD. Patients are expected to adhere to long‐term pharmacological treatment, risk factor control and lifestyle modification after discharge [5]. Thus, post‐PCI rehabilitation extends beyond the in‐hospital treatment episode; rather, discharge marks the beginning of a long‐term phase centred on self‐management, secondary prevention and continuous care.

Discharge marks a critical transition from hospital‐based treatment to home‐based recovery for patients following PCI. With advances in interventional cardiology and increasingly short hospital stays after uncomplicated PCI, responsibilities for medication management, symptom monitoring, lifestyle modification, follow‐up attendance and cardiac rehabilitation are rapidly transferred to patients and their caregivers. Evidence suggests that the hospital‐to‐home transition is associated with postdischarge healthcare utilisation, including readmission, and that transitional care interventions may improve these outcomes to some extent [6]. Therefore, discharge readiness should encompass patients’ understanding of their disease and treatment, recognition of warning symptoms, adherence to medical advice, access to social support and engagement in subsequent rehabilitation [7]. A previous study of patients with CHD after stent implantation found that discharge readiness was associated with treatment adherence within 30 days of discharge, suggesting that discharge readiness assessment may help identify potential risks related to treatment adherence and self‐management [8]. Therefore, systematic predischarge assessment of discharge readiness among patients following PCI may help identify deficits in disease knowledge, symptom recognition, treatment implementation and rehabilitation support, thereby informing individualised discharge guidance, nurse‐led transitional care and continuity of care.

Discharge readiness has commonly been assessed using generic instruments, among which the Readiness for Hospital Discharge Scale (RHDS) is one of the most widely used [9]. The RHDS assesses patients’ perceived readiness to return home after acute hospitalisation and covers domains such as personal status, knowledge, coping ability and expected support, thereby providing an overall evaluation of patients’ readiness for discharge. However, the RHDS was not designed for a specific disease or treatment modality. When applied to patients with CHD following PCI, the RHDS may not fully capture PCI‐specific preparation needs, including medication management and adherence, puncture‐site care, recognition of warning symptoms, timely help‐seeking, risk‐factor management, and cardiac rehabilitation. A recent scoping review similarly reported that existing discharge‐readiness instruments are primarily used for general discharge assessment in acutely hospitalised patients and may capture readiness from either the patient or the nurse perspective; however, the contextual applicability of their content across different clinical settings requires further attention [10]. Although previous studies have assessed discharge readiness and its associated factors among patients after PCI for myocardial infarction or coronary stent implantation, most have used generic instruments and have focused primarily on levels of discharge readiness, influencing factors, or treatment adherence [8, 11, 12]. These studies provide useful evidence but offer limited guidance for nurses conducting structured predischarge assessments and identifying specific support needs before patients return home. To our knowledge, a PCI‐specific nurse‐assessed instrument tailored to the discharge transition of patients with CHD following PCI has not yet been developed. Therefore, there remains a need to develop a PCI‐specific nurse‐assessed discharge readiness scale for patients with CHD to support standardised discharge planning and transitional care management.

Transition theory, proposed by Meleis et al. [13], explains how individuals adapt to changes in health status, roles, relationships and care environments, and highlights the influence of transition conditions, response patterns and nursing interventions on health outcomes. During the discharge process after PCI, patients experience rapid changes in health status, care environment and self‐management roles. Their level of readiness may influence postdischarge rehabilitation adaptation and the need for continuity of care. Using the transition theory framework, discharge readiness among patients with CHD following PCI was conceptualised, and scale items were developed to construct the nurse‐assessed Discharge Readiness Scale for Patients with CHD Following Percutaneous Coronary Intervention (DRS‐CHD‐PCI). Therefore, this study aimed to develop the DRS‐CHD‐PCI based on transition theory and conduct its initial psychometric validation. The scale was designed to provide nurses with a structured approach to identifying areas in which patients may require further preparation before discharge and to inform individualised discharge education and nurse‐led transitional care planning following PCI.

## 2. Methods

### 2.1. Study Design

A multistage design was used to develop and conduct the initial psychometric validation of the nurse‐assessed DRS‐CHD‐PCI, comprising item development, Delphi consultation, content validity assessment, pilot testing and psychometric validation in a clinical sample. This process followed recommended procedures for scale development and validation in health, social and behavioural research [14].

### 2.2. Item Development

The conceptual framework for item generation was guided by transition theory. The research team conducted a targeted review of relevant literature, clinical guidelines and existing discharge‐readiness instruments covering discharge readiness, secondary prevention of CHD, rehabilitation after PCI, cardiac rehabilitation and transitional care. Candidate items relevant to discharge readiness among patients with CHD following PCI were then extracted.

Semistructured interviews were conducted to supplement the perspectives of patients and healthcare professionals on discharge readiness after PCI. Purposive sampling was used to recruit patients with CHD who had undergone PCI from the cardiology wards of a tertiary Grade A hospital in Sichuan Province, south‐western China. Cardiologists and cardiovascular nurses were also invited to participate in the interviews. Patients were eligible if they: (1) had been diagnosed with CHD by a cardiologist and had undergone PCI; (2) were aged 18 years or older; (3) were clinically stable, conscious and able to understand and communicate effectively and (4) agreed to participate and provided written informed consent. Patients were excluded if they: (1) had a diagnosed mental disorder or cognitive impairment; (2) were unable to complete the interview or declined audio recording or (3) had severe complications or unstable clinical conditions. In total, 15 patients with CHD following PCI and 6 healthcare professionals, comprising 4 cardiovascular nurses and 2 cardiologists, participated in the semistructured interviews. Detailed demographic and professional characteristics of the interview participants are presented in Supporting S1.

The patient interview outline focused on the impact of hospitalisation on daily life and emotional status, difficulties with post‐discharge rehabilitation, support and knowledge needs for disease recovery and emergency self‐management, and concerns about self‐management after returning home. The healthcare professionals’ interview outline focused on key aspects of inpatient observation after PCI, priorities for health education, discharge criteria, discharge guidance and factors influencing discharge readiness. Audio recordings were transcribed and analysed using Colaizzi’s seven‐step method [15]. The researchers repeatedly reviewed the transcripts, extracted significant statements, coded and categorised the text, and then aggregated similar codes into emergent themes. NVivo 11 was used to facilitate data organisation and coding. The credibility of the qualitative analysis was enhanced through research team discussions and member checking.

The research team integrated the theoretical framework, findings from the literature and guidelines, existing discharge‐readiness instruments and interview analysis results to generate candidate scale items. These items were then evaluated for theoretical relevance, PCI‐specific relevance, clinical importance, clarity of wording and patient comprehensibility and feasibility for structured nurse assessment. Items with overlapping content, similar wording or limited clinical operability were merged, revised or deleted. The resulting initial item pool was then submitted for Delphi expert consultation.

### 2.3. Delphi Consultation and Content Validity Assessment

For the Delphi consultation, purposive sampling was used to invite experts in cardiovascular clinical medicine, cardiovascular nursing and nursing management to serve on the expert panel. Experts were eligible if they: (1) had at least 10 years’ experience in cardiovascular clinical practice, nursing, nursing management or related research; (2) held a bachelor’s degree or higher; (3) held an intermediate or higher professional title; (4) were familiar with the management of patients following PCI or with discharge readiness; and (5) were willing to participate in the study and complete the Delphi consultation.

The Delphi consultation was conducted between May and July 2022, with two rounds held at 2‐3‐week intervals. After each round, the research team summarised experts’ ratings and open‐ended comments and revised the consultation questionnaire for the subsequent round. Questionnaires were distributed and returned online or in paper format in both rounds. The expert consultation questionnaire comprised an introduction to the study, an evaluation form for the scale dimensions and items and a demographic information form for the experts. Experts rated the importance of each dimension and item on a 5‐point Likert scale, with 1 indicating ‘*not important*’ and 5 indicating ‘*very important*’. Open‐ended comment sections were also provided to allow experts to suggest changes to item wording, dimensional allocation, item addition or deletion, and clinical applicability. Expert engagement, authority and agreement were evaluated using the questionnaire response rate, expert authority coefficient, coefficient of variation and Kendall’s W coefficient of concordance. Item selection was based on expert ratings, open‐ended comments, content representativeness and research team discussions.

A total of 17 experts completed both rounds of the Delphi consultation. Following the revision of the items based on the consultation, an initial 23‐item scale was developed.

After two rounds of Delphi consultation and item revision, eight additional healthcare professionals with more than 10 years’ experience in cardiology were invited to assess the content validity of the 23‐item preliminary scale. The raters evaluated each item’s relevance to the construct of discharge readiness among patients with CHD following PCI, using a 4‐point rating scale, with 1 indicating ‘not relevant’ and 4 indicating ‘highly relevant’. Ratings of 3 or 4 were considered indicative of item relevance. Content validity was assessed using the item‐level content validity index (I‐CVI) and the average scale‐level content validity index (S‐CVI/Ave). An I‐CVI of 0.78 or higher and an S‐CVI/Ave of 0.90 or higher were considered acceptable [16].

### 2.4. Pilot Test

Before formal psychometric validation, the research team conducted a pilot test to assess the comprehensibility, acceptability and feasibility of the 23‐item preliminary nurse‐assessed scale in the target population and for structured nurse assessment. The pilot assessment form comprised a demographic information form and the initial version of the DRS‐CHD‐PCI. Using convenience sampling, 20 eligible patients with CHD following PCI were recruited from the cardiology wards of a tertiary Grade A hospital in Sichuan Province, south‐western China. Nurses administered the preliminary scale through structured assessment and collected feedback from patients on the comprehensibility of the assessment content and from nurses on item wording, scoring criteria, response options and completion time. The pilot test showed that patients understood and accepted the assessment content well and that nurses considered the scoring criteria clinically feasible. The assessment took approximately 10–15 min to complete, and both the scale length and item wording were considered feasible. Therefore, no substantial modifications were made to the scale items.

### 2.5. Psychometric Validation

For psychometric validation, convenience sampling was used to recruit patients with CHD who had undergone PCI from the cardiology wards of a tertiary Grade A hospital in Sichuan Province, south‐western China, between August 2022 and February 2023. Patients were eligible if they: (1) had been diagnosed with CHD by a cardiologist and had undergone PCI; (2) were aged 18 years or older; (3) were clinically stable, conscious and able to understand and communicate effectively; (4) were scheduled for discharge on the day of the assessment and (5) were willing to participate and provided written informed consent. Patients were excluded if they: (1) had a diagnosed mental disorder or cognitive impairment or (2) were unable to provide the information required for the structured nurse assessment because of impaired consciousness, communication difficulties or unstable clinical conditions.

The preliminary DRS‐CHD‐PCI was completed by nurses as a structured predischarge assessment on the planned day of discharge. Nurses assessed each patient using information from patient responses, clinical observations and relevant clinical assessment results. The assessment covered psychological status, chest pain severity, puncture‐site wound healing, activities of daily living, emergency response ability, symptom recognition, caregiver support, access to healthcare resources, adherence‐related awareness and disease‐related knowledge. The nurse assessment took approximately 10–15 min to complete.

The preliminary scale developed after the Delphi consultation contained 23 items. Methodological guidance for factor analysis recommends a sample size of at least seven times the number of items, with no fewer than 100 participants in each analysis [17]. On this basis, the minimum target sample size was 161 participants per analysis. A total of 460 patients were approached to participate in the structured nurse assessment, and 444 valid assessments were retained for analysis (96.5%). Using computer‐generated random numbers, the final sample was divided into two independent subsamples. Subsample 1 (*n* = 215) was used for item analysis and exploratory factor analysis, whereas Subsample 2 (*n* = 229) was used for confirmatory factor analysis and reliability analyses of the final scale. Both subsamples exceeded the recommended minimum.

The response formats were operationalised using a common five‐point ordinal metric ranging from 1 to 5, with higher scores indicating greater discharge readiness. In the final 17‐item version, Item 1 used criterion‐referenced scoring anchors based on the Generalized Anxiety Disorder 7‐item scale (GAD‐7; [18]) and the 9‐item Patient Health Questionnaire (PHQ‐9; [19]). Item 2 used the 11‐point Numeric Rating Scale for pain, and Item 4 used the Barthel Index. The remaining items used ordered verbal response options. For Item 1, the GAD‐7 and PHQ‐9 scores were converted separately, and the lower of the two converted scores was recorded as the item score. Raw scores from these clinical instruments were not directly included in the scale total. Following item reduction, the 17 retained item scores were summed without weighting to produce a total score ranging from 17 to 85. No clinical cut‐off was applied to classify patients as ready or not ready. The complete scoring anchors and conversion rules are presented in Supporting S7.

### 2.6. Data Analysis

Statistical analyses were conducted using SPSS version 27.0 and AMOS version 24.0. Continuous variables were summarised with means and standard deviations, and categorical variables with frequencies and percentages. A two‐sided *p* value < 0.05 was considered statistically significant.

#### 2.6.1. Item Analysis

Item quality was evaluated using the critical ratio method, Spearman item–total correlation analysis and homogeneity testing. Homogeneity testing included corrected item–total correlations and changes in Cronbach’s *α* following item deletion. Decisions on item deletion were based on statistical performance, theoretical relevance, clinical interpretability and construct representativeness.

#### 2.6.2. Structural Validity

Exploratory factor analysis was conducted on Subsample 1 to examine the scale’s underlying factor structure. Before factor analysis, the Kaiser–Meyer–Olkin test and Bartlett’s test of sphericity were used to assess the data’s suitability for factor analysis. Principal component analysis with varimax rotation and Kaiser normalisation was used to explore the factor structure. The number of factors and item retention were determined by considering eigenvalues, the scree plot, factor loadings, communalities, cross‐loadings and theoretical interpretability. In general, items with high primary factor loadings, acceptable communalities and no substantial cross‐loadings were preferentially retained.

Confirmatory factor analysis was conducted on Subsample 2 to assess the stability of the factor structure identified by exploratory factor analysis. Based on the exploratory factor analysis results and the theoretical structure, first‐ and second‐order confirmatory factor analysis models were specified. Model fit was evaluated using standard fit indices, including the chi‐square‐to‐degrees‐of‐freedom ratio (*χ* ^2^/df), comparative fit index (CFI), Tucker–Lewis index (TLI), root mean square error of approximation (RMSEA) and standardised root mean square residual (SRMR) [20]. Convergent validity was assessed using the average variance extracted (AVE) and composite reliability (CR). Discriminant validity was evaluated by comparing the square root of the AVE for each dimension with the correlations between dimensions [21].

#### 2.6.3. Reliability

Internal consistency reliability was evaluated using Cronbach’s *α*. Split‐half reliability was computed to assess consistency across the two halves of the scale.

### 2.7. Ethical Considerations

This study was approved by the Ethics Committee of the Sichuan Academy of Medical Sciences & Sichuan Provincial People’s Hospital (Approval No. 2022‐96). All participants provided written informed consent before data collection. Qualitative interviews were audio‐recorded only after participants’ permission was obtained. All interview materials and assessment data were anonymised, and no personally identifiable information was included in the reporting of the study findings. The study was conducted in accordance with the Declaration of Helsinki.

### 2.8. Use of Artificial Intelligence Tools

During manuscript preparation, ChatGPT was used to assist with English‐language editing and the refinement of academic expression. The authors reviewed, revised and approved all AI‐assisted text and take full responsibility for the manuscript’s content. No artificial intelligence tool was used to generate research data, conduct statistical analyses, interpret results or make authorship decisions.

## 3. Results

### 3.1. Delphi Consultation Results

The initial item pool submitted for Delphi consultation comprised five candidate dimensions and 58 candidate items. A total of 17 experts completed both rounds of the Delphi consultation. Characteristics of the Delphi experts and the consultation results are presented in Supporting S2. Based on expert ratings and open‐ended comments, the research team systematically merged, revised, deleted or added items to the original 58‐item pool. After two rounds of Delphi consultation, the item pool was reduced from 58 to 23 items, forming the preliminary version of the scale for subsequent pilot testing and psychometric validation.

### 3.2. Content Validity

Based on the initial 23‐item scale developed after two Delphi rounds, the content validity assessment showed that the I‐CVI ranged from 0.875 to 1.000, and the S‐CVI/Ave was 0.941. These findings indicated that the preliminary 23‐item scale had acceptable item‐ and scale‐level content validity.

### 3.3. Characteristics of the Participants

A total of 444 patients with CHD who had undergone PCI were included in the psychometric validation phase. Their demographic characteristics are presented in Table 1. Participants aged ≥ 70 years accounted for the largest proportion of the sample (*n* = 248, 55.86%), followed by those aged 60–69 years (*n* = 128, 28.83%). Most participants were male (*n* = 327, 73.65%), married, living in urban areas and living with their children.

**TABLE 1:** Demographic and clinical characteristics of the psychometric validation sample (*n* = 444).

Variable	Category	*n*	%
Age group, years	< 50	22	4.95
	50–59	46	10.36
	60–69	128	28.83
	≥ 70	248	55.86

Sex	Female	117	26.35
	Male	327	73.65

Marital status	Married	422	95.05
	Unmarried	4	0.90
	Widowed	18	4.05

Educational level	Primary school or below	68	15.32
	Junior high school or senior high school	236	53.15
	College or above	140	31.53

Employment status	Self‐employed	87	19.59
	Farmers	76	17.12
	Employees	63	14.19
	Retired	218	49.10

Place of residence	Rural	117	26.35
	Urban	327	73.65

Living arrangement	Living alone	20	4.50
	Living with a spouse	122	27.48
	Living with children	302	68.02

Number of comorbidities	0	90	20.27
	1	248	55.86
	2	86	19.37
	≥ 3	20	4.50

Body mass index, kg/m^2^	< 18.5	10	2.25
	18.5–23.9	56	12.61
	24.0–27.9	260	58.56
	≥ 28.0	118	26.58

### 3.4. Item Analysis

Item analysis indicated that several items showed relatively weak discrimination, low corrected item–total correlations or an increase in Cronbach’s *α* upon item deletion. Based on prespecified statistical criteria and item‐specific theoretical and clinical considerations, six items (Items 2, 3, 8, 11, 20 and 22) were deleted from the 23‐item preliminary scale. Although Items 8 and 11 met the discrimination criteria, their corrected item–total correlations were 0.144 and 0.197, respectively, both below the criterion of 0.30, and their deletion increased Cronbach’s *α* from 0.706 to 0.830 and from 0.687 to 0.813, respectively. Item 8 also overlapped conceptually with Item 7 and had limited applicability to routine home recovery, whereas Item 11 framed social support primarily as passive help‐seeking and was potentially confusing for older patients. The scale was therefore reduced from 23 to 17 items and subsequently subjected to exploratory factor analysis. Detailed item analysis results and item‐specific reasons for deletion are presented in Supporting S3.

### 3.5. Structural Validity

#### 3.5.1. Exploratory Factor Analysis

An EFA was conducted on Subsample 1 (*n* = 215) to examine the exploratory factor structure. The Kaiser–Meyer–Olkin (KMO) value was 0.928, and Bartlett’s test of sphericity was statistically significant (*χ* ^2^ = 2457.187, df = 136, *p* < 0.001), indicating that the data were suitable for factor analysis. Based on the theoretical dimensional structure and the scree plot (Supporting S4), five factors were extracted, explaining 75.843% of the total variance. Primary factor loadings for all retained items exceeded 0.50, and the retained items showed interpretable loading patterns. The final scale comprised 17 items across five dimensions. Based on item content and primary factor loadings, the five dimensions were named Psychological and Physical Condition, Self‐Care and Coping Ability, Social Support, Adherence and Disease‐Related Knowledge. Primary factor loadings and item communalities are presented in Table 2, and the final scale items and response options are provided in Supporting S7.

**TABLE 2:** Exploratory factor analysis loadings and communalities of the final 17‐item scale in Subsample 1 (*n* = 215).

No.	Items	Factor 1	Factor 2	Factor 3	Factor 4	Factor 5	Communality
1	Psychological status before discharge	0.239	**0.772**	0.250	0.161	0.112	0.753
2	Chest pain severity	0.207	**0.775**	0.150	0.117	0.257	0.745
3	Puncture‐site wound healing	0.168	**0.752**	0.210	0.263	0.193	0.744
4	Ability to perform activities of daily living	0.207	0.404	0.093	0.256	**0.698**	0.768
5	Ability to take basic emergency actions	0.332	0.148	0.318	0.209	**0.694**	0.759
6	Recognition of symptoms requiring medical attention	0.399	0.234	0.263	0.217	**0.667**	0.775
7	Caregiver support for medication use	0.191	0.238	0.242	**0.715**	0.289	0.747
8	Access to medical and transitional care services	0.161	0.232	0.167	**0.828**	0.140	0.812
9	Access to medical and disease‐related information	0.346	0.097	0.263	**0.678**	0.155	0.682
10	Awareness of the importance of medication adherence	0.258	0.185	**0.824**	0.204	0.197	0.861
11	Awareness of the importance of timely medical consultation	0.310	0.376	**0.649**	0.291	0.154	0.767
12	Awareness of the importance of continuing transitional care	0.244	0.247	**0.768**	0.274	0.226	0.836
13	Knowledge of medication effects and side effects	**0.714**	0.153	0.315	0.132	0.319	0.752
14	Knowledge of a heart‐healthy diet	**0.643**	0.280	0.381	0.234	0.263	0.762
15	Knowledge of the FITT principle for exercise	**0.713**	0.141	0.169	0.220	0.222	0.653
16	Knowledge of emotional regulation and sleep management	**0.713**	0.377	0.116	0.148	0.149	0.708
17	Knowledge of disease‐related risk factors	**0.816**	0.126	0.168	0.198	0.134	0.767
Eigenvalue		3.451	2.636	2.469	2.305	2.032	
Variance explained (%)		20.299	15.508	14.522	13.561	11.953	
Cumulative variance explained (%)		20.299	35.807	50.329	63.889	75.843	

*Note:* Factor 1 = disease‐related knowledge; Factor 2 = psychological and physical condition; Factor 3 = adherence; Factor 4 = social support; Factor 5 = self‐care and coping ability. The bold values indicate the highest factor loading for each item and identify the factor to which the item was assigned.

#### 3.5.2. Confirmatory Factor Analysis

CFA was conducted on Subsample 2 (*n* = 229) to assess the five‐dimensional structure identified by EFA. Both the first‐ and second‐order models showed acceptable fit. The core fit indices for the first‐order model were *χ* ^2^/df = 2.097, CFI = 0.958, TLI = 0.948, RMSEA = 0.070 and SRMR = 0.038. For the second‐order model, the corresponding values were *χ* ^2^/df = 2.173, CFI = 0.954, TLI = 0.954, RMSEA = 0.072 and SRMR = 0.043. The standardised loadings of the five first‐order dimensions on the higher‐order discharge‐readiness factor ranged from 0.810 to 0.977. Detailed second‐order factor loadings are presented in Supporting S5.2, and first‐order item loadings are provided in Supporting S5.3. The full set of model fit indices is presented in Table 3, and the path diagram for the second‐order model is shown in Supporting S5.1.

**TABLE 3:** Model fit indices for confirmatory factor analysis in Subsample 2 (*n* = 229).

Model	*χ* ^2^	*χ* ^2^/df	CFI	TLI	RMSEA	SRMR	NFI
Recommended criteria		< 3	≥ 0.90	≥ 0.90	≤ 0.08	≤ 0.08	> 0.90
First‐order model	228.546	2.097	0.958	0.948	0.070	0.038	0.924
Second‐order model	247.686	2.173	0.954	0.954	0.072	0.043	0.918

Abbreviations: *χ* ^2^ = chi‐square statistic; *χ* ^2^/df = chi‐square statistic divided by degrees of freedom; CFI = comparative fit index; df = degrees of freedom; NFI = normed fit index’ RMSEA = root mean square error of approximation; SRMR = standardised root mean square residual; TLI = Tucker–Lewis index.

#### 3.5.3. Convergent and Discriminant Validity

Convergent validity analysis showed that the AVE values for the five dimensions ranged from 0.630 to 0.735, and the CR values ranged from 0.836 to 0.899, all of which met acceptable criteria. Discriminant validity analysis showed that the square root of the AVE for each dimension exceeded its correlations with the other dimensions, supporting acceptable discriminant validity of the scale. Detailed results are presented in Supporting S6.

### 3.6. Reliability

The overall Cronbach’s *α* for the final scale was 0.945, and the Cronbach’s *α* for the five dimensions ranged from 0.813 to 0.894. The split‐half reliability for the total scale was 0.896, and the Guttman split‐half coefficient was 0.895. These findings indicate satisfactory internal consistency and split‐half reliability. Reliability results for each dimension are presented in Table 4.

**TABLE 4:** Reliability coefficients of the final 17‐item scale and its dimensions in Subsample 2 (*n* = 229).

Dimension	Number of items	Cronbach’s *α*
Psychological and physical condition	3	0.836
Self‐care and coping ability	3	0.830
Social support	3	0.813
Adherence	3	0.889
Disease‐related knowledge	5	0.894
Total scale	17	0.945

*Note:* The split‐half reliability coefficient and Guttman split‐half coefficient for the total scale were 0.896 and 0.895, respectively.

## 4. Discussion

This study identified a five‐dimensional structure of discharge readiness among patients with CHD following PCI, comprising Psychological and Physical Condition, Self‐Care and Coping Ability, Social Support, Adherence, and Disease‐Related Knowledge. Transition theory provides a framework for interpreting these dimensions in relation to the conditions shaping transition and patients’ awareness, engagement and responses as they assume new self‐management responsibilities [13]. Psychological and Physical Condition reflects personal conditions that may affect patients’ participation in the hospital‐to‐home transition. Self‐Care and Coping Ability reflects developing confidence and coping, along with emerging mastery of daily self‐care, emergency response and symptom recognition. Social Support encompasses interpersonal and community‐level resources: caregiver support reflects feeling connected, while access to medical and transitional care services and disease‐related information reflects community‐level transition conditions. Adherence captures patients’ awareness of behaviours needed to support the transition, including medication adherence, timely medical consultation and continued engagement with transitional care. Disease‐Related Knowledge corresponds to preparation and knowledge. Together, the five dimensions translate selected elements of transition theory into clinically relevant content for assessing preparation for the hospital‐to‐home transition following PCI.

The multidimensional structure is consistent with a previous concept analysis, which identified physical stability, psychological ability, adequate support and adequate information and knowledge as defining attributes of readiness for hospital discharge [7]. Building on this broader conceptualisation, the DRS‐CHD‐PCI may be viewed as a PCI‐specific extension of existing discharge‐readiness assessment for cardiovascular populations. For example, the Readiness for Hospital Discharge after Myocardial Infarction Scale comprises three subscales assessing patients’ subjective knowledge, objective knowledge and expectations regarding discharge [22]. In contrast, the DRS‐CHD‐PCI incorporates content on puncture‐site recovery, symptom recognition, emergency response and timely help‐seeking, access to transitional care, exercise‐based rehabilitation, psychological and sleep‐related concerns and risk‐factor awareness. These items overlap with several core components emphasised in the 2024 scientific statement from the American Heart Association and the American Association of Cardiovascular and Pulmonary Rehabilitation, including patient assessment, nutritional counselling, cardiovascular disease and risk‐factor management, psychosocial management, aerobic exercise training and physical activity counselling [23]. Accordingly, the main contribution of the DRS‐CHD‐PCI is not to redefine discharge readiness but to translate this generic construct into PCI‐specific, nurse‐assessed content that may support structured discharge assessment and transitional care planning.

The reliability and factor‐analytic findings provide preliminary support for the internal consistency and structural validity of the DRS‐CHD‐PCI. However, because the scale is nurse‐assessed, differences in how nurses interpret and apply the scoring criteria may introduce measurement variation and limit comparability of scores across assessors and healthcare settings.

Beyond these measurement considerations, symptom improvement at discharge does not necessarily indicate adequate preparation for home‐based recovery among patients following PCI. Previous research has shown that long‐term self‐management after coronary stent implantation remains suboptimal, emphasising the need for continued attention to disease management and risk‐reduction behaviours after discharge [24]. Therefore, pre‐discharge assessment should consider not only symptom relief and medical stability but also patients’ preparedness for home‐based recovery. The dimension scores provide a descriptive profile of readiness across the domains of psychological and physical condition, self‐care and coping ability, social support, adherence‐related awareness and disease‐related knowledge. However, whether lower total or dimension scores are associated with adverse post‐discharge outcomes remains unknown. Establishing predictive validity is therefore a critical next step. Longitudinal studies are needed to examine these associations before the scale can be recommended for outcome prediction or clinical decision‐making.

## 5. Implications for Nursing Management

The DRS‐CHD‐PCI may provide nursing managers and cardiovascular nursing teams with a structured approach to predischarge assessment following PCI. In routine practice, the scale may help nurses move beyond general discharge education and highlight specific areas in which patients may require additional support, such as symptom recognition, medication adherence, puncture‐site observation, rehabilitation knowledge and access to transitional care services. At the service level, dimension‐specific scores may provide descriptive information to inform discharge education, follow‐up planning and nurse‐led transitional care. However, these potential applications were not evaluated in the present study. Before broader implementation, prospective multicentre studies should examine scoring consistency across nurses, predictive validity and whether use of the scale improves patient and service outcomes.

## 6. Limitations

This study has several limitations. First, the psychometric validation sample was recruited from a single tertiary Grade A hospital in south‐western China using convenience sampling, which may limit the generalisability of the findings to other regions and healthcare institutions at different levels. The sample may also have been relatively homogeneous in terms of patient characteristics and care contexts, which may have affected the stability of the factor structure and potentially inflated the proportion of variance explained. Therefore, the five‐factor structure should be interpreted cautiously pending replication in more diverse samples. Second, this study developed the DRS‐CHD‐PCI and conducted an initial psychometric validation. Consistent with the COSMIN framework, further evaluation is needed to assess additional measurement properties, including test–retest reliability, measurement error and responsiveness [25]. Inter‐rater reliability was not evaluated; therefore, scoring consistency across nurses remains unknown. A dedicated inter‐rater reliability study is planned as a subsequent validation step. Moreover, combining criterion‐referenced ordinal scores (including those for Items 1 and 4) with Likert‐type item scores in an equally weighted total assumes comparable spacing across response categories. This assumption was not empirically tested; future Rasch analyses should examine category threshold ordering and spacing. Third, this study did not examine relationships between scale scores and postdischarge outcomes, such as treatment adherence, participation in cardiac rehabilitation, unplanned readmission or health‐related quality of life. In addition, because the study did not include an established external criterion or prospective clinical outcome, a clinically validated cut‐off value for distinguishing adequate from inadequate discharge readiness could not be established. Accordingly, the DRS‐CHD‐PCI should currently be interpreted as a continuous measure rather than used for binary classification. Future multicentre, longitudinal studies involving more diverse samples from hospitals at different levels of care and across geographical regions are needed to evaluate further the generalisability, predictive validity, responsiveness, scoring consistency across nurses and clinical utility of the DRS‐CHD‐PCI, and to establish clinically meaningful thresholds using external criteria and relevant postdischarge outcomes.

## 7. Conclusion

The DRS‐CHD‐PCI is a 17‐item, five‐dimensional nurse‐assessed scale designed to assess discharge readiness as a continuous construct among patients with CHD following PCI. This study provides preliminary evidence supporting its content validity, structural validity and internal consistency. Further evaluation of its stability, responsiveness, predictive validity and clinical utility in larger, multicentre longitudinal studies across diverse populations, healthcare settings and cultural contexts is required before broader clinical use can be recommended.

## Author Contributions

Xingxing Mo: conceptualisation, methodology, investigation, formal analysis, writing–original draft; Ting Liang: conceptualisation, methodology, investigation, data curation, writing–review and editing; Juejin Li: supervision, project administration, writing–review and editing; Xuan Wang: conceptualisation, supervision, project administration, writing–review and editing.

## Funding

No funding has been received for this paper.

## Ethics Statement

This study was approved by the Ethics Committee of the Sichuan Academy of Medical Sciences & Sichuan Provincial People’s Hospital (Approval No. 2022‐96). All participants provided written informed consent before data collection. The study was conducted in accordance with the Declaration of Helsinki.

## Conflicts of Interest

The authors declare no conflicts of interest.

## Supporting Information

Additional supporting information can be found online in the Supporting Information section.

## Supporting information


**Supporting Information** Supporting information is available online.

## Data Availability

The data that support the findings of this study are available from the corresponding author upon reasonable request.
