# Supplementary material for: Development and Initial Psychometric Validation of a Nurse‐Assessed Discharge Readiness Scale for Patients With Coronary Heart Disease Following Percutaneous Coronary Intervention
Source: J Nurs Manag. 2026 Oct 19;2026:5080347. doi: 10.1155/jonm/5080347 (PMC13678577; doi:10.1155/jonm/5080347)
Supplement: Supplementary file 1 [file JONM-2026-5080347-s001.docx]

**Supplementary S1. Characteristics of interview participants**

**S1.1 Demographic characteristics of interviewed patients with CHD following PCI (n = 15)**

| **No.** | **Sex** | **Age (years)** | **Education level** | **Occupational status** | **Living arrangement** | **Marital status** | **Place of residence** |
| --- | --- | --- | --- | --- | --- | --- | --- |
| P1 | Male | 77 | Primary school | Farmer | Living with spouse and children | Married | Urban |
| P2 | Male | 58 | Junior college | Employed | Living with spouse and children | Married | Urban |
| P3 | Male | 78 | Primary school | Retired | Living with spouse and children | Married | Urban |
| P4 | Male | 52 | Junior high school | Employed | Living with spouse | Married | Rural |
| P5 | Female | 70 | Primary school | Farmer | Living with spouse | Married | Rural |
| P6 | Male | 81 | Senior high school | Retired | Living with children | Widowed | Urban |
| P7 | Male | 65 | Junior college | Retired | Living with spouse | Married | Urban |
| P8 | Female | 57 | Junior high school | Employed | Living with spouse | Married | Urban |
| P9 | Male | 56 | Senior high school | Employed | Living with spouse and children | Married | Urban |
| P10 | Male | 57 | Junior college | Employed | Living with spouse and children | Married | Urban |
| P11 | Male | 45 | Bachelor's degree | Employed | Living with spouse and children | Married | Urban |
| P12 | Female | 70 | Primary school | Retired | Living with spouse | Married | Urban |
| P13 | Male | 41 | Junior high school | Farmer | Living with parents | Single | Rural |
| P14 | Male | 68 | Junior college | Retired | Living with spouse and children | Married | Urban |
| P15 | Female | 65 | Junior college | Retired | Living with spouse | Married | Urban |

***Note.*** *P = patient*

**S1.2 Demographic and professional characteristics of interviewed healthcare professionals (n = 6)**

| **No.** | **Sex** | **Age (years)** | **Professional experience (years)** | **Highest educational level** | **Professional title** | **Professional role** |
| --- | --- | --- | --- | --- | --- | --- |
| M1 | Female | 58 | 40 | Bachelor's degree | Senior | Cardiovascular nurse |
| M2 | Female | 53 | 34 | Bachelor's degree | Associate senior | Cardiovascular nurse |
| M3 | Female | 36 | 12 | Master's degree | Intermediate | Cardiovascular nurse |
| M4 | Female | 35 | 11 | Bachelor's degree | Intermediate | Cardiovascular nurse |
| M5 | Male | 53 | 28 | Master's degree | Senior | Cardiologist |
| M6 | Male | 45 | 20 | Doctoral degree | Associate senior | Cardiologist |

***Note.*** *M = healthcare professional participant. Professional titles are reported according to the Chinese professional title classification system.*

**Supplementary S2. Characteristics of the Delphi experts and results of the Delphi consultation**

**S2.1. Characteristics of the Delphi experts (n = 17)**

| Variable | Category | *n* | % |
| --- | --- | --- | --- |
| Sex | Female | 3 | 17.65 |
|  | Male | 14 | 82.35 |
| Age group, years | 30–39 | 3 | 17.65 |
|  | 40–49 | 6 | 35.29 |
|  | ≥50 | 8 | 47.06 |
| Years of working experience | 10–19 | 4 | 23.53 |
|  | 20–29 | 5 | 29.41 |
|  | ≥30 | 8 | 47.06 |
| Postgraduate supervisor | Yes | 5 | 29.41 |
|  | No | 12 | 70.59 |
| Professional title | Intermediate | 5 | 29.41 |
|  | Associate senior | 6 | 35.29 |
|  | Senior | 6 | 35.29 |
| Highest educational level | Bachelor’s degree | 13 | 76.48 |
|  | Master’s degree | 2 | 11.76 |
|  | Doctoral degree | 2 | 11.76 |
| Research field | Clinical cardiovascular medicine | 3 | 17.65 |
|  | Cardiovascular nursing management | 5 | 29.41 |
|  | Clinical cardiovascular nursing practice | 9 | 52.94 |

**S2.2. Results of the two rounds of Delphi expert consultation**

| **Round / Level** | **Response rate (%)** | **Experts with comments (%)** | **Authority coefficient** | ***CV*** | ***Kendall’s W*** | ***χ²*** | ***df*** | ***P*** |
| --- | --- | --- | --- | --- | --- | --- | --- | --- |
| Round 1 overall | 100 | 64.71 | 0.856 | 0.144 | 0.242 | 255.532 | 62 | <0.001 |
| Dimensions | — | — | — | 0.122 | 0.211 | 14.349 | 4 | <0.001 |
| Items | — | — | — | 0.145 | 0.244 | 236.632 | 57 | <0.001 |
| Round 2 overall | 100 | 17.65 | 0.871 | 0.091 | 0.272 | 152.561 | 33 | <0.001 |
| Dimensions | — | — | — | 0.074 | 0.390 | 26.545 | 4 | <0.001 |
| Items | — | — | — | 0.085 | 0.256 | 121.708 | 28 | <0.001 |

***Note.*** *CV = coefficient of variation; Kendall’s W = Kendall’s coefficient of concordance; χ² = chi-square statistic; df = degrees of freedom; P = probability value.*

**Supplementary S3. Item analysis results and item-specific reasons for deletion**

| **Dimension** | **Item** | **CR** | **Spearman I–T correlation** | **CITC** | **α if item deleted** | **Decision and item-specific rationale** |
| --- | --- | --- | --- | --- | --- | --- |
| Dimension 1 | Item 1 | 18.878 | 0.758 | 0.622 | 0.486 | Retained |
|  | Item 2 | **0.792** | **0.230** | — | — | Deleted—A fixed blood-pressure threshold was inconsistent with individualised treatment targets. |
|  | Item 3 | 4.115 | 0.407 | **0.028** | **0.836** | Deleted—LDL-C targets were individualised, and patients had difficulty recalling baseline values and personal targets. |
|  | Item 4 | 15.674 | 0.756 | 0.652 | 0.473 | Retained |
|  | Item 5 | 15.810 | 0.737 | 0.621 | 0.503 | Retained |
| Dimension 2 | Item 6 | 15.284 | 0.743 | 0.527 | 0.623 | Retained |
|  | Item 7 | 22.683 | 0.833 | 0.652 | 0.535 | Retained |
|  | Item 8 | 6.612 | 0.465 | **0.144** | **0.830** | Deleted—The item overlapped conceptually with Item 7 and had limited applicability to routine home recovery. |
|  | Item 9 | 22.253 | 0.866 | 0.717 | 0.490 | Retained |
| Dimension 3 | Item 10 | 18.944 | 0.816 | 0.633 | 0.512 | Retained |
|  | Item 11 | 8.204 | 0.568 | **0.197** | **0.813** | Deleted—The item framed social support primarily as passive help-seeking and was potentially confusing for older patients. |
|  | Item 12 | 16.142 | 0.808 | 0.648 | 0.521 | Retained |
|  | Item 13 | 17.330 | 0.731 | 0.505 | 0.601 | Retained |
| Dimension 4 | Item 14 | 28.851 | 0.912 | 0.799 | 0.828 | Retained |
|  | Item 15 | 24.314 | 0.890 | 0.754 | 0.867 | Retained |
|  | Item 16 | 26.240 | 0.911 | 0.795 | 0.831 | Retained |
| Dimension 5 | Item 17 | 23.159 | 0.821 | 0.736 | 0.845 | Retained |
|  | Item 18 | 25.683 | 0.866 | 0.803 | 0.833 | Retained |
|  | Item 19 | 19.100 | 0.781 | 0.692 | 0.853 | Retained |
|  | Item 20 | 10.463 | 0.631 | **0.456** | **0.894** | Deleted—The item overlapped with other lifestyle-related knowledge items; Delphi experts recommended deletion to reduce redundancy. |
|  | Item 21 | 15.397 | 0.775 | 0.699 | 0.853 | Retained |
|  | Item 22 | **2.636** | **0.259** | — | — | Deleted—The item had limited response acceptability and was not considered universally applicable to patients following PCI. |
|  | Item 23 | 18.406 | 0.804 | 0.735 | 0.847 | Retained |

***Note.*** *Dimensions: 1 = Psychological and Physical Condition; 2 = Self-Care and Coping Ability; 3 = Social Support; 4 = Adherence; 5 = Disease-Related Knowledge. CR = critical ratio; I–T = item–total; CITC = corrected item–total correlation; LDL-C = low-density lipoprotein cholesterol; PCI = percutaneous coronary intervention; α = Cronbach’s alpha; — = not calculated because the item was excluded before homogeneity testing. Deletion decisions considered prespecified statistical criteria, theoretical relevance, clinical applicability, and content redundancy.*

**Supplementary S4. Results of exploratory factor analysis**

**Figure S4. Scree plot from the exploratory factor analysis of the DRS-CHD-PCI.**

***Note.*** *DRS-CHD-PCI = Discharge Readiness Scale for Patients with Coronary Heart Disease Following Percutaneous Coronary Intervention.*

**Supplementary S5. Results of confirmatory factor analysis**

**Figure S5.1 Second-order confirmatory factor analysis path diagram of the DRS-CHD-PCI.**

***Note.*** *Values represent standardised factor loadings. CFA = confirmatory factor analysis; DRS-CHD-PCI = Discharge Readiness Scale for Patients with Coronary Heart Disease Following Percutaneous Coronary Intervention.*

**S5.2 Second-order factor loadings of the five dimensions on the DRS-CHD-PCI**

| **First-order dimension** | **Unstandardised estimate** | **Standardised estimate** | **S.E.** | **C.R.** | **p** |
| --- | --- | --- | --- | --- | --- |
| Psychological and Physical Condition | 1.000 | 0.822 | — | — | — |
| Self-Care and Coping Ability | 0.898 | 0.907 | 0.083 | 10.776 | <0.001 |
| Social Support | 0.840 | 0.810 | 0.085 | 9.910 | <0.001 |
| Adherence | 1.055 | 0.870 | 0.094 | 11.286 | <0.001 |
| Disease-Related Knowledge | 1.082 | 0.977 | 0.088 | 12.289 | <0.001 |

*Note. DRS-CHD-PCI = Discharge Readiness Scale for Patients with Coronary Heart Disease Following Percutaneous Coronary Intervention; S.E. = standard error; C.R. = critical ratio. The unstandardised loading of Psychological and Physical Condition was fixed at 1.000 for model identification; — = not estimated.*

**S5.3 First-order factor loadings of items on their respective dimensions**

| **Item** | **Dimension** | **Unstandardised estimate** | **Standardised estimate** | **S.E.** | **C.R.** | **p** |
| --- | --- | --- | --- | --- | --- | --- |
| N1 | Psychological and Physical Condition | 1.000 | 0.868 | — | — | — |
| N4 | Psychological and Physical Condition | 0.789 | 0.774 | 0.059 | 13.479 | <0.001 |
| N5 | Psychological and Physical Condition | 0.791 | 0.818 | 0.054 | 14.533 | <0.001 |
| N6 | Self-Care and Coping Ability | 1.000 | 0.795 | — | — | — |
| N7 | Self-Care and Coping Ability | 1.091 | 0.781 | 0.087 | 12.515 | <0.001 |
| N9 | Self-Care and Coping Ability | 1.054 | 0.821 | 0.079 | 13.292 | <0.001 |
| N10 | Social Support | 1.000 | 0.807 | — | — | — |
| N12 | Social Support | 0.882 | 0.803 | 0.069 | 12.829 | <0.001 |
| N13 | Social Support | 1.038 | 0.822 | 0.079 | 13.151 | <0.001 |
| N14 | Adherence | 1.000 | 0.865 | — | — | — |
| N15 | Adherence | 0.882 | 0.855 | 0.053 | 16.513 | <0.001 |
| N16 | Adherence | 0.961 | 0.868 | 0.057 | 16.926 | <0.001 |
| N17 | Disease-Related Knowledge | 1.000 | 0.854 | — | — | — |
| N18 | Disease-Related Knowledge | 1.088 | 0.890 | 0.060 | 18.059 | <0.001 |
| N19 | Disease-Related Knowledge | 0.821 | 0.712 | 0.065 | 12.549 | <0.001 |
| N21 | Disease-Related Knowledge | 0.865 | 0.791 | 0.059 | 14.735 | <0.001 |
| N23 | Disease-Related Knowledge | 0.885 | 0.789 | 0.060 | 14.660 | <0.001 |

***Note.*** *S.E. = standard error; C.R. = critical ratio. Item identifiers refer to the original 23-item preliminary scale. The first unstandardised loading within each dimension was fixed at 1.000 for model identification; — = not estimated.*

**Supplementary S6. Results of construct validity analyses**

**S6.1 Convergent validity indices**

| Dimension | AVE | CR |
| --- | --- | --- |
| Psychological and physical condition | 0.656 | 0.851 |
| Self-care and coping ability | 0.631 | 0.837 |
| Social support | 0.630 | 0.836 |
| Adherence | 0.735 | 0.893 |
| Disease-related knowledge | 0.642 | 0.899 |

***Note.*** *AVE = average variance extracted; CR = composite reliability.*

**S6.2 Discriminant validity indices**

| Dimension | Dimension 1 | Dimension 2 | Dimension 3 | Dimension 4 | Dimension 5 |
| --- | --- | --- | --- | --- | --- |
| Dimension 1 | 0.810 |  |  |  |  |
| Dimension 2 | 0.637 | 0.795 |  |  |  |
| Dimension 3 | 0.615 | 0.607 | 0.794 |  |  |
| Dimension 4 | 0.621 | 0.680 | 0.632 | 0.858 |  |
| Dimension 5 | 0.647 | 0.753 | 0.658 | 0.725 | 0.801 |

***Note.*** *Dimension 1 = psychological and physical condition; Dimension 2 = self-care and coping ability; Dimension 3 = social support; Dimension 4 = adherence; Dimension 5 = disease-related knowledge. Diagonal values represent the square roots of AVE calculated from unrounded estimates, and off-diagonal values represent correlations between dimensions.*

**Supplementary S7. Final version of the DRS-CHD-PCI scale**

**Scoring.** All 17 items are scored on a common five-point ordinal metric ranging from 1 to 5, with higher scores indicating greater discharge readiness. Item 1 uses criterion-referenced clinical anchors based on the GAD-7 and PHQ-9; Item 2 uses the 11-point Numeric Rating Scale for pain; and Item 4 uses the Barthel Index. The remaining items use ordered verbal response anchors. Item scores are summed without weighting to produce a total score ranging from 17 to 85. No clinically validated cut-off is currently available; scores should therefore be interpreted as a continuous measure rather than used to classify patients as ready or not ready.

**Item 1 rule.** GAD-7 and PHQ-9 scores are categorised separately using the thresholds shown below. If the resulting categories differ, the more severe category (i.e., the lower item score) is used as the final Item 1 score. Because the standard GAD-7 comprises four severity categories, an Item 1 score of 2 can be assigned only based on a PHQ-9 score of 15–19.

| **Item** | **Response options and scoring anchors** |
| --- | --- |
| **Dimension 1. Psychological and Physical Condition** | |
| Item 1. Psychological status before discharge | 1 = Severe (GAD-7: 15–21; PHQ-9: 20–27)  2 = Moderately severe (GAD-7: not applicable; PHQ-9: 15–19)  3 = Moderate (GAD-7: 10–14; PHQ-9: 10–14)  4 = Mild (GAD-7: 5–9; PHQ-9: 5–9)  5 = Minimal (GAD-7: 0–4; PHQ-9: 0–4) |
| Item 2. Chest pain severity | 1 = Severe pain (NRS = 10)  2 = Moderate-to-severe pain (NRS = 7–9)  3 = Moderate pain (NRS = 4–6)  4 = Mild pain (NRS = 1–3)  5 = No pain (NRS = 0) |
| Item 3. Puncture-site wound healing | 1 = Strongly disagree  2 = Disagree  3 = Neutral  4 = Agree  5 = Strongly agree |
| **Dimension 2. Self-Care and Coping Ability** | |
| Item 4. Ability to perform activities of daily living | 1 = Total dependence (Barthel Index: <20)  2 = Severe dependence (Barthel Index: 20–40)  3 = Moderate dependence (Barthel Index: 41–60)  4 = Slight dependence (Barthel Index: 61–99)  5 = Independence (Barthel Index: 100) |
| Item 5. Ability to take basic emergency actions | 1 = Not familiar at all  2 = Slightly familiar  3 = Moderately familiar  4 = Mostly familiar  5 = Fully familiar |
| Item 6. Recognition of symptoms requiring medical attention | 1 = Not familiar at all  2 = Slightly familiar  3 = Moderately familiar  4 = Mostly familiar  5 = Fully familiar |
| **Dimension 3. Social Support** | |
| Item 7. Caregiver support for medication use | 1 = Not familiar at all  2 = Slightly familiar  3 = Moderately familiar  4 = Mostly familiar  5 = Fully familiar |
| Item 8. Access to medical and transitional care services | 1 = Not familiar at all  2 = Slightly familiar  3 = Moderately familiar  4 = Mostly familiar  5 = Fully familiar |
| Item 9. Access to medical and disease-related information | 1 = Not familiar at all  2 = Slightly familiar  3 = Moderately familiar  4 = Mostly familiar  5 = Fully familiar |
| **Dimension 4. Adherence** | |
| Item 10. Awareness of the importance of medication adherence | 1 = Not familiar at all  2 = Slightly familiar  3 = Moderately familiar  4 = Mostly familiar  5 = Fully familiar |
| Item 11. Awareness of the importance of timely medical consultation | 1 = Not familiar at all  2 = Slightly familiar  3 = Moderately familiar  4 = Mostly familiar  5 = Fully familiar |
| Item 12. Awareness of the importance of continuing transitional care | 1 = Not familiar at all  2 = Slightly familiar  3 = Moderately familiar  4 = Mostly familiar  5 = Fully familiar |
| **Dimension 5. Disease-Related Knowledge** | |
| Item 13. Knowledge of medication effects and side effects | 1 = Not familiar at all  2 = Slightly familiar  3 = Moderately familiar  4 = Mostly familiar  5 = Fully familiar |
| Item 14. Knowledge of a heart-healthy diet | 1 = Not familiar at all  2 = Slightly familiar  3 = Moderately familiar  4 = Mostly familiar  5 = Fully familiar |
| Item 15. Knowledge of the FITT principle for exercise | 1 = Not familiar at all  2 = Slightly familiar  3 = Moderately familiar  4 = Mostly familiar  5 = Fully familiar |
| Item 16. Knowledge of emotional regulation and sleep management | 1 = Not familiar at all 2 = Slightly familiar 3 = Moderately familiar 4 = Mostly familiar 5 = Fully familiar |
| Item 17. Knowledge of disease-related risk factors | 1 = Not familiar at all  2 = Slightly familiar  3 = Moderately familiar  4 = Mostly familiar  5 = Fully familiar |

***Note.*** *DRS-CHD-PCI = Discharge Readiness Scale for Patients with Coronary Heart Disease Following Percutaneous Coronary Intervention; FITT = frequency, intensity, time, and type; GAD-7 = Generalized Anxiety Disorder 7-item scale; NRS = 11-point Numeric Rating Scale for pain (0 = no pain; 10 = worst pain imaginable); PHQ-9 = 9-item Patient Health Questionnaire.*
